# Macrophage targeting: opening new possibilities for cancer immunotherapy

**DOI:** 10.1111/imm.12976

**Published:** 2018-07-31

**Authors:** Luca Cassetta, Takanori Kitamura

**Affiliations:** ^1^ MRC Centre for Reproductive Health Queen's Medical Research Institute The University of Edinburgh Edinburgh UK; ^2^ Royal (Dick) School of Veterinary Studies and the Roslin Institute The University of Edinburgh Edinburgh UK

**Keywords:** cancer, immunotherapy, macrophage, tumour immunology

## Abstract

Tumour‐infiltrating immune cells regulate tumour development and progression either negatively or positively. For example, cytotoxic lymphocytes (CTL) such as CD8^+^ T and natural killer (NK) cells can recognize and eliminate cancer cells, and thereby restrict the tumour growth and metastasis, if they exert full cytotoxicity. In contrast, tumour‐infiltrating myeloid cells such as tumour‐associated macrophages (TAM) promote the expansion and dissemination of cancer cells depending on their functional states. Given the tumour‐killing ability of CTL, the augmentation of CTL‐induced antitumour immune reactions has been considered as an attractive therapeutic modality for lethal solid tumours and several promising strategies have emerged, which include immune checkpoint inhibitors, cancer vaccines and adoptive CTL transfer. These immunotherapies are now tested in clinical trials and have shown significant antitumour effects in patients with lymphoma and some solid tumours such as melanoma and lung cancer. Despite these encouraging results, these therapies are not efficient in a certain fraction of patients and tumour types with tumour cell‐intrinsic mechanisms such as impaired antigen presentation and/or tumour cell‐extrinsic mechanisms including the accumulation of immunosuppressive cells. Several animal studies suggest that tumour‐infiltrating myeloid cells, especially TAM, are one of the key targets to improve the efficacy of immunotherapies as these cells can suppress the functions of CD8^+^ T and NK cells. In this review, we will summarize recent animal studies regarding the involvement of TAM in the immune checkpoint, cancer vaccination and adoptive CTL transfer therapies, and discuss the therapeutic potential of TAM targeting to improve the immunotherapies.

**Abbreviations**
ARG1: arginase‐1
CAR: chimeric antigen receptor
CCR: CC‐chemokine receptor
CEA: carcinoembryonic antigen
CSF1R: colony‐stimulating factor 1 receptor
CTLA4: cytotoxic T‐lymphocyte‐associated protein 4
CTL: cytotoxic lymphocyte
DC: dendritic cell
FASL: first apoptosis signal ligand
Fc*γ*R: Fc‐*γ* receptor
FR*β*: folate receptor *β*
IL: interleukin
LLC: Lewis lung carcinoma
MARCO: macrophage receptor with collagenous structure
MHC‐I: major histocompatibility complex class I
NK: natural killer
PD1: programmed cell death protein 1
PI3K*γ*: phosphoinositide 3‐kinase *γ*
PyMT: polyoma middle T oncogene
TAM: tumour‐associated macrophage
TGF‐*β*: transforming growth factor‐*β*
TLR: toll‐like receptor
TNF‐*α*: tumour necrosis factor‐*α*

## Introduction

Solid tumours are initiated by oncogenic mutations in non‐haematopoietic cells and progress into lethal tumour masses that account for 80% of all mortality of patients with cancer.^1^ Cytotoxic lymphocytes (CTL) such as CD8^+^ T and natural killer (NK) cells are critical for suppressing the development and progression of lethal solid tumours, as these cells can eliminate tumour cells once they exert full cytotoxicity.^2^ Therefore, many efforts have been invested to maximize the tumoricidal abilities of CTLs by understanding CTL regulatory mechanisms and developing CTL engineering technologies, which gives rise to attractive therapeutic modalities – for example, immunotherapies such as cancer vaccination, immune checkpoint therapy and adoptive CTL transfer therapy.

For effective killing of tumour cells, CD8^+^ T cells require largely three steps. At first, antigen‐presenting cells such as dendritic cells (DC) take up and process immunogenic aberrant proteins produced by genetic mutations in tumour cells (i.e. neoantigens) and present them to naive CD8^+^ T cells in the lymph node. Second, the primed and activated CD8^+^ T cells expand clonally and migrate into the tumours. At last, the effector CD8^+^ T cells recognize the antigenic peptides presented on the surface of tumour cells by major histocompatibility complex class I (MHC‐I) and transmit apoptotic signals into the tumour cells.^3^ Cancer vaccination and immune checkpoint therapy are developed to amplify this endogenous antitumour response^3^ and so are suitable for targeting immunogenic tumours that present neoantigens to CD8^+^ T cells. However, these therapies are not ideal to eliminate tumour cells that do not express tumour antigens or MHC‐I. To eliminate this type of cancer cell, adoptive transfer of CTL such as NK cells or engineered T cells has emerged. Unlike CD8^+^ T cells, NK cells do not require MHC‐I‐mediated priming or prior activation to kill their target cells. Instead, the cytotoxicity of NK cells is regulated by the balance of signals from inhibitory receptors and activating receptors on their surface. Tumour cells often up‐regulate NK activating receptor ligands and lose NK inhibitory receptor ligands, including MHC‐I molecules,^4^ and so this type of cancer cells can be eliminated by NK cells, whereas endogenous CD8^+^ T cells do not recognize them. Adoptive transfer of genetically engineered T cells that directly recognize cell surface proteins on tumour cells has also been developing as another attractive strategy to target such non‐immunogenic tumour cells.^5^

It is therefore likely that proper selection of therapeutic strategies is important to improve the outcome of immunotherapy, that is monotherapies using cancer vaccine or checkpoint inhibitors for immunogenic tumours and CTL transfer therapies for non‐immunogenic tumours. On the other hand, removal of immune suppressive factors in the tumour microenvironment, along with efficient CTL delivery, has been suggested as key issues to design effective immunotherapies for solid tumours. Most solid tumours include a variety of immune cells such as regulatory T cells, myeloid‐derived suppressor cells and tumour‐associated macrophages (TAM) that can suppress CTL functions.^6^, ^7^ Among these cells, TAM is one of the most abundant cell types in solid tumours^8^ and their infiltration into the tumour associates with poor prognosis in most solid tumours.^9^, ^10^, ^11^, ^12^, ^13^, ^14^ Furthermore, macrophages isolated from mouse and human solid tumours can directly suppress T‐cell responses^15^, ^16^ and NK cell cytotoxicity^17^, ^18^ *in vitro*. It is also reported that depletion of TAM enhances CD8^+^ T‐cell‐mediated antitumour immunity under treatment with chemotherapy in a mouse model of breast cancer.^19^ In addition, results from other mouse models of breast cancer indicate that physical contact of TAM with tumour‐infiltrating CD8^+^ T cells suppresses full activation of T cells or their access to the tumour cells.^20^, ^21^ Therefore, TAM has been suggested as one of the important therapeutic targets to enhance the efficacy of immunotherapy.^22^

**Figure 1:**
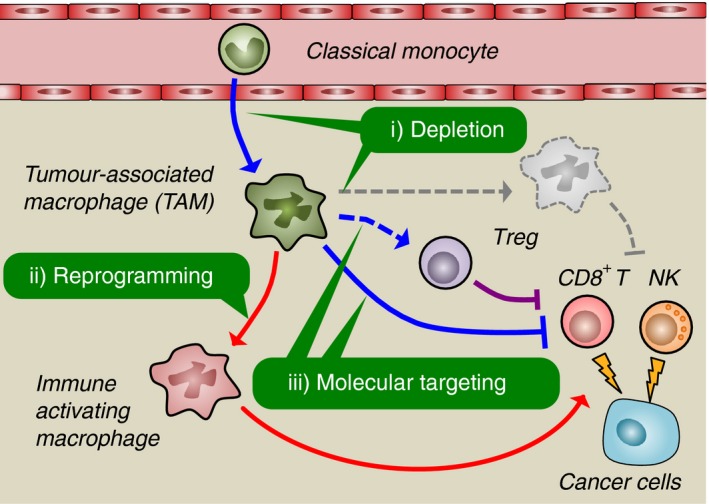
Potential therapeutic approaches to prevent tumour‐associated macrophage (TAM) ‐mediated immune suppression. Classical monocytes in the blood are recruited to the solid tumours where they differentiate to TAM. The TAM suppress cytotoxicity of CD8^+^ T or natural killer (NK) cells directly via expressing immune suppressive molecules or indirectly via the recruitment of other immune suppressor cells such as regulatory T (Treg) cells. However, TAM can become immune‐activating macrophages in response to certain environmental factors. It is therefore likely that either prevention of TAM accumulation (depletion), alteration of TAM features (reprogramming), or blockade of TAM‐derived immune suppressive molecules (molecular targeting) are attractive approaches to dislodge the TAM‐induced immune suppressive environment in the solid tumours.

To prevent the TAM‐mediated immune suppression, there are at least three therapeutic approaches, that is depletion, reprogramming and molecular targeting (Fig. 1). It has been suggested that the continuous accumulation of TAM requires the recruitment of circulating monocytes that differentiate into TAM in the tumour.^22^ In mouse models of breast cancer, the recruitment of monocytes into the tumour microenvironment is promoted via the CC‐chemokine receptor 2 (CCR2), and hence, blockade of CCR2 can suppress the accumulation of TAM in the tumours.^23^, ^24^, ^25^ It is also well known that colony‐stimulating factor 1 receptor (CSF1R) signal is essential for the recruitment, differentiation and survival of macrophages, and the loss of CSF1/CSF1R dramatically reduces the number of TAM in mouse models of solid tumour.^22^, ^26^, ^27^ Therefore, blockade of signals for monocyte recruitment and/or macrophage survival can reduce the TAM accumulation in tumours and thereby dislodge the immune suppressive tumour microenvironment. Another approach to block immune suppression by TAM is reprogramming of their characteristics. It is now widely accepted that plasticity is a hallmark of macrophages. For example, macrophages cultured with interleukin‐4 (IL‐4) and IL‐13 (called alternatively activated macrophages) produce anti‐inflammatory cytokines such as IL‐10 and transforming growth factor‐*β* (TGF‐*β*) that can inhibit CD8^+^ T‐cell functions, whereas macrophages cultured with lipopolysaccharide and interferon‐*γ* (called classically activated macrophages) secrete pro‐inflammatory cytokines such as tumour necrosis factor‐*α* (TNF‐*α*) and IL‐1*β*.^28^ Furthermore, alternatively activated macrophages can change their phenotype to classically activated macrophages when they are exposed to interferon‐*γ* and lipopolysaccharide.^29^ As alternatively but not classically activated macrophages suppress *in vitro* T‐cell proliferation,^30^ these *in vitro* studies suggest that targeting macrophage differentiation signals can reprogram TAM from immune suppressive to supportive cells and thereby enhance antitumour immune reactions induced by immunotherapy. Although the precise mechanisms behind TAM‐mediated immune suppression are still unclear, several studies suggest that TAM can suppress T‐cell activities directly via expression of arginase‐1 (ARG1), IL‐10 and TGF‐*β*, as well as indirectly through recruitment of other immune suppressor cells like regulatory T cells.^31^ Targeting these molecules can be another attractive approach to eliminate TAM‐mediated restriction of immunotherapy.

## TAM targeting for immune checkpoint therapy

**Figure 2:**
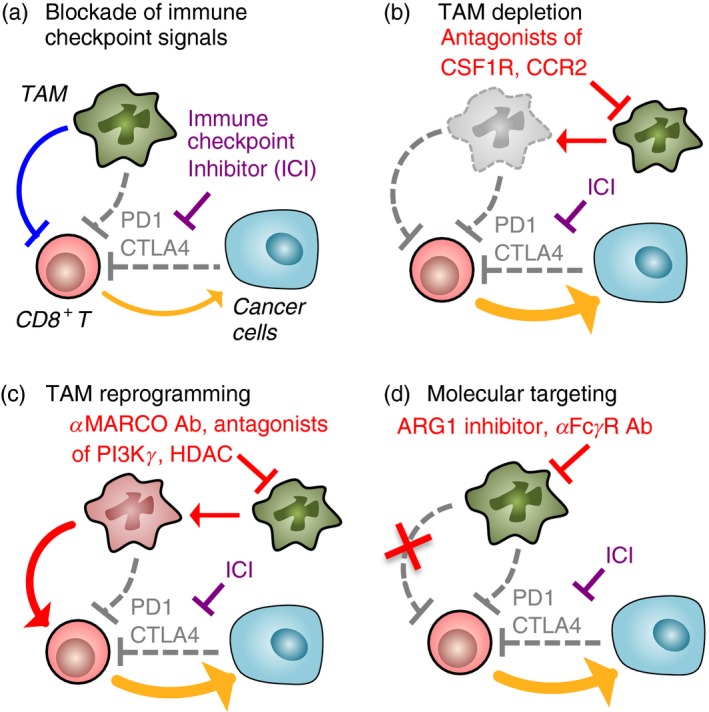
Effects of tumour‐associated macrophages (TAM) ‐targeting strategies on efficacy of immune checkpoint inhibitors. Immune checkpoint inhibitor (ICI) enhances CD8^+^ T‐cell cytotoxicity by blocking immune checkpoint pathways via programmed cell death protein 1 (PD1) or cytotoxic T‐lymphocyte‐associated protein 4 (CTLA4) activated by cancer cells and TAM. However, TAM also suppresses CD8^+^ T‐cell functions via checkpoint pathway‐independent mechanisms and limits the efficacy of ICI (a). Several studies suggest that all potential TAM‐targeting approaches, that is TAM depletion (b), TAM reprogramming (c), and targeting functional molecules of TAM (d), can improve the therapeutic efficacy of ICI. The yellow arrow represents cytotoxicity of CD8^+^ T cells.

To demonstrate strong antitumour responses, effector T cells need to overcome intrinsic negative regulation pathways transmitted via immune checkpoint receptors such as programmed cell death protein 1 (PD1) or cytotoxic T‐lymphocyte‐associated protein 4 (CTLA4). In many cases, cancer cells and stromal cells express ligands for PD1 (PD‐L1, PD‐L2) and/or CTLA4 (CD80, CD86) and thereby restrict the tumoricidal abilities of CD8^+^ T cells. To overcome this restriction, administration of blocking antibodies against these checkpoint receptors/ligands (checkpoint inhibitors) has been tested in clinical trials and has shown dramatic therapeutic effects in patients with melanoma and lung cancer.^32^ However, the majority of patients with other types of solid tumours, such as pancreatic and breast cancer, do not fully respond to this type of immunotherapy,^33^, ^34^ probably because large numbers of solid tumours including pancreatic and breast cancer (but not melanoma and lung cancer) have a low mutation rate that reduces the opportunity of neoantigen expression to be recognized by CD8^+^ T cells.^35^ The excluded immune cell infiltration can be another profile of non‐responding tumours where CD8^+^ T cells remain outside the tumours (i.e. peritumoral stroma) and fail to reach and eliminate tumour cells.^36^ Indeed, a recent study demonstrated that urothelial cancers in patients who did not respond to an anti‐PDL1 agent showed a low tumour mutation rate and a preferential accumulation of CD8^+^ T cells in the fibroblast‐rich peritumoral stroma rather than the tumour parenchyma.^37^ Another potential reason why checkpoint inhibitors show poor activity is because myeloid cells in the tumour microenvironment, especially TAM, limit the cytotoxicity of the activated CD8^+^ T cells by unknown mechanisms (Fig. 2a).^38^

In a mouse model of pancreatic cancer, treatment with a CCR2 antagonist decreases the infiltration of monocyte/macrophage in the tumour. In this model, CCR2 antagonist treatment in combination with anti‐PD1 antibody suppresses tumour growth, whereas single treatment with anti‐PD1 antibody is not effective.^39^ Therefore, it is likely that blockade of CCR2 signalling prevents TAM accumulation in the tumour and thereby enhances the efficacy of immune checkpoint inhibition. Treatment with CSF1R antagonists (e.g. PLX3397, PLX73086 and BLZ945) also markedly reduces the number of TAM in mouse models of mesothelioma, and of ovarian, cervical, breast and pancreatic cancers.^19^, ^40^, ^41^, ^42^, ^43^ In the mouse model of pancreatic cancer, treatment of tumour‐bearing mice with a CSF1R antagonist PLX3397 in combination with anti‐PD1 and anti‐CTLA4 antibodies results in the complete blockade of tumour expansion, although single treatment with PLX3397 or anti‐PD1/anti‐CTLA4 shows only modest suppression of tumour growth.^43^ A recent study also shows that the depletion of macrophages by PLX3397 in combination with anti‐PD1 therapy increases the accumulation of CD8^+^ T cells in the tumour and delays tumour progression in a mouse model of breast cancer.^21^ In mouse models of melanoma, single treatment with anti‐PD1 antibodies can suppress tumour growth, although several mice do not respond to the treatment. In contrast, combined treatment with anti‐PD1 and anti‐CSF1R antibodies induces tumour regression in all mice and prolongs their survival.^44^ These results indicate that the TAM depletion by targeting CCR2 and/or CSF1R can improve the efficacy of checkpoint inhibitors (Fig. 2b).

Recent studies suggest that TAM reprogramming can also enhance the antitumour effects of checkpoint inhibitors. In mice with melanoma established by subcutaneous injection of B16 cells, administration of neutralizing antibody against macrophage receptor with collagenous structure (MARCO), a scavenger receptor predominantly expressed by TAM, enhances the efficacy of anti‐CTLA4 antibody treatment in suppressing tumour growth.^45^ As the treatment of anti‐MARCO antibody reduces IL‐10 expression and concomitantly increases IL‐1*β* expression in TAM, these results suggest that targeting MARCO can switch the TAM phenotype from immunosuppressive (alternatively activated) to immune activating (classically activated) and thereby promote antitumour activities of cytotoxic T cells. Inhibition of phosphoinositide 3‐kinase *γ* (PI3K*γ*) signalling may also change the immune suppressive phenotype of TAM as genetic deletion of PI3K*γ* gene (*Pik3cg*) reduces the expression of ARG1, IL‐10 and TGF‐*β* in cultured alternatively activated macrophages.^46^ The loss of *Pik3cg* also reduces *Arg1*,* Il10* and *Tgfb* mRNA expression in TAM and enhances the cytotoxicity of T cells in the subcutaneous tumours established by Lewis lung carcinoma (LLC) cells, suggesting that blockade of PI3K*γ* signalling promotes the antitumour effects of T‐cell‐based immunotherapies by blocking immune suppressive functions of TAM. In line with this notion, a PI3K*γ* inhibitor (TG100‐15) markedly enhances the tumour suppressive effects of anti‐PD1 antibody in a mouse model of head and neck squamous carcinoma.^46^ In the mammary tumours developed in polyoma middle T oncogene (PyMT) transgenic mice, a selective class IIa histone deacetylase inhibitor (TMP195) alters predominant macrophage populations in the tumour from TAM to highly phagocytic macrophages. In this model, administration of TMP195 combined with anti‐PD1 antibody significantly suppresses tumour development, whereas a single treatment with TMP195 or anti‐PD1 antibody shows modest suppression of the tumour burden.^47^ Therefore, targeting master regulators of macrophage differentiation (e.g. MARCO, PI3K*γ* and histone deacetylase) can be a potential approach to enhance checkpoint therapy by harnessing immune suppressive features and/or drawing antitumour functions in tumour‐infiltrating macrophages (Fig. 2c).

It is well known that alternatively activated macrophages express high levels of ARG1, an l‐arginine processing enzyme that can suppress T‐cell functions by depleting l‐arginine from the environment.^31^ It is also reported that TAM isolated from the subcutaneous tumours established by C3 fibrosarcoma or LLC cells express high levels of ARG1 and suppress T‐cell proliferation via ARG1‐mediated mechanisms.^48^, ^49^ In mice that have received orthotopic injection of 4T1 mammary tumour cells, the treatment with anti‐PD1/anti‐CTLA4 antibodies combined with an ARG1 inhibitor (CB‐1158) significantly suppresses primary tumour growth and lung metastases.^50^ Likewise, treatment with CB‐1158 enhances the tumour suppressive effect of anti‐PD‐L1 antibody in mice with subcutaneous tumours developed by CT26 colon cancer cells.^45^ These results highlight the possibility that molecular targeting of TAM‐derived factors can be another approach to prevent TAM‐mediated restriction of checkpoint therapy (Fig. 2d). Although further studies are needed to identify targetable molecules that are expressed by TAM to suppress T‐cell cytotoxicity, a recent study suggests Fc*γ* receptor (Fc*γ*R), a receptor of immunoglobulin, as a candidate. In subcutaneous tumours established by MC38 colon cancer cells, anti‐PD1 antibodies injected into the mice initially bind to T cells but are deprived by TAM via Fc*γ*R within 24 hr. Furthermore, injection of anti‐PD1 antibody with Fc*γ*R‐blocking antibody completely suppresses tumour growth in all mice whereas the response to a single anti‐PD1 treatment varies among animals,^51^ suggesting that targeting the interaction of Fc receptors in TAM with the Fc region of checkpoint blocking antibodies can improve the therapeutic efficacy of checkpoint inhibitors. These studies in mouse models confirm that all three TAM‐targeting strategies (depletion, reprogramming and molecular targeting) could potentially be used in combination with checkpoint inhibitors to synergistically improve the response to this kind of immunotherapy.

## TAM targeting for cancer vaccination

An additional approach to boost CD8^+^ T‐cell reactions against tumours is cancer vaccination. Vaccination involves the administration of antigenic materials aimed at the stimulation of the endogenous immune system with the final goal to prevent the onset of infections and tumours (preventive vaccination) or treat the diseases (therapeutic vaccination). Preventive vaccinations, where the antigens are injected before the disease takes place, aim to ‘simulate’ a bacterial or a viral infection so that the immune system, specifically B cells, can generate antibodies against the pathogen and memory for the potential real encounter with the pathogen. On the other side, therapeutic vaccinations aim to educate naive CD8^+^ T cells to become cytotoxic lymphocytes through antigen presentation by antigen‐presenting cells such as DC and thereby activate the immune system against an ongoing infection or neoplastic lesion that the immune system was not able to detect. Although this therapy is advantageous in cost, ease and safety, its therapeutic response is so far quite limited, probably because each tumour has different mutations, and hence, neoantigens expressed by tumour cells are diverse. However, recent advances in deep‐sequencing technologies enable us to identify the mutations in an individual tumour and thereby predict optimal neoantigens to prime and activate CD8^+^ T cells.^35^ It has also been suggested that efficacy of cancer vaccination can be enhanced by co‐injection of immune adjuvants that amplify the host immune responses such as toll‐like receptor (TLR) ligands and DC‐targeted antibodies.^52^ Another approach for the refinement of this type of therapy is the *ex vivo* generated DC‐based vaccines where DC cultured with whole tumour cell lysate or antigenic peptide are injected back into patients.^53^ Advances in all of these components will make therapeutic vaccination more efficient. As in other immunotherapies, however, recent studies have demonstrated that the efficacy of cancer vaccination is strongly linked with the level of accumulation and activation of myeloid cells, especially macrophages.

**Figure 3:**
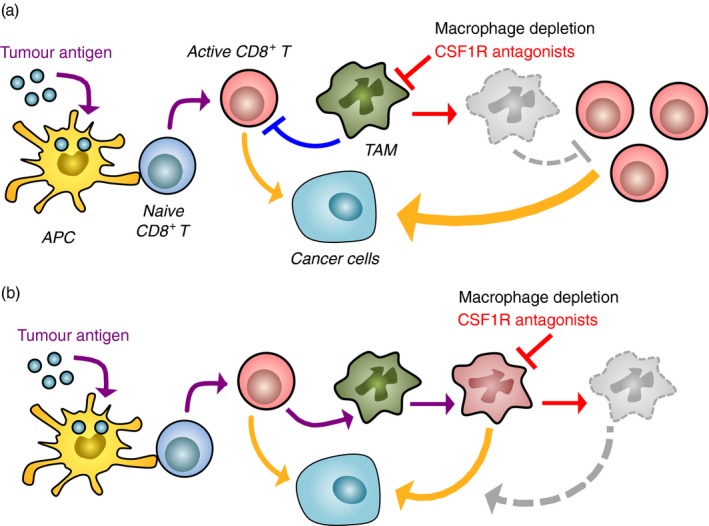
Opposite effects of macrophage depletion on therapeutic cancer vaccination therapy. Administration of tumour antigen with strong adjuvant or dendritic cells pre‐exposed to antigen activates naive CD8^+^ T cells that exert antitumour ability. However, their cytotoxicity is suppressed by tumour‐associated macrophages (TAM), and hence, pharmacological macrophage depletion, for example treatment with a colony‐stimulating factor 1 receptor (CSF1R) antagonist, enhances efficacy of vaccinations (a). In some cases, however, the activated CD8^+^ T cells alter the phenotype of TAM from immune suppressive to immune‐activating/tumoricidal. Under such situations, macrophage depletion reduces rather than enhances the efficacy of therapeutic cancer vaccination (b). The yellow arrow represents cytotoxicity of CD8^+^ T cells or reprogrammed macrophages.

For example, injection of tumour lysate‐pulsed DC (DC‐based vaccination) prolongs survival of mice that have been orthotopically injected with syngeneic mesothelioma cells, and this therapeutic effect is further enhanced by DC‐based vaccination in combination with injection of PLX3397, a CSF1R inhibitor that depletes macrophages.^40^ Depletion of TAM also enhances the efficacy of therapeutic vaccination with strong adjuvants. In a murine model of ovarian cancer, immunization with microparticles containing ligands of TLR9 and nucleotide‐binding oligomerization domain 2 leads to the accumulation of T cells in the tumours and prolongs the survival of tumour‐bearing mice. On the other hand, the vaccination also increases accumulation of T‐cell suppressive CD11b^+^ myeloid cells in the peritoneum.^54^ In this model, CD11b‐mediated depletion of myeloid cells shows a synergistic effect in combination with the vaccine by further prolonging the survival of tumour‐bearing mice even if no significant reduction in tumour size was observed.^54^ Similar results are reported in a mouse model of cervical cancer (i.e. subcutaneous injection of TC‐1 cancer cells) in which tumour‐bearing mice are immunized with a di‐palmitoylated peptide, a self‐adjuvanting antigen that stimulates DC maturation and primes CTLs in a TLR2/6‐specific fashion.^55^ In this model, depletion of myeloid cells by clodronate liposome injection increases the efficacy of the lipopeptide‐based vaccination in reducing tumour size and prolonging survival of tumour‐bearing mice (Fig. 3a). However, macrophage depletion is not always associated with an improved response to vaccination even in the similar cervical tumour mouse model. A recent study shows that injection of a long peptide with incomplete Freund's adjuvant to mice that have established the subcutaneous TC‐1 cervical tumours induces a significant accumulation of CD8^+^ T cells as well as macrophages in the tumour lesion. Interestingly, vaccination‐induced tumour regression in this case is abrogated rather than enhanced by macrophage depletion before and during the peptide injection using the inhibitor PLX3397, suggesting that the reprogramming of resident macrophages or the recruitment of pro‐inflammatory macrophages by vaccination is responsible for therapeutic efficacy (Fig. 3b).^56^ These studies suggest that differences in the vaccination protocol affects features of macrophages in the tumours, and hence, TAM‐targeting strategies should be carefully evaluated to combine with cancer vaccination.

## TAM targeting for adoptive CTL transfer therapy

**Figure 4:**
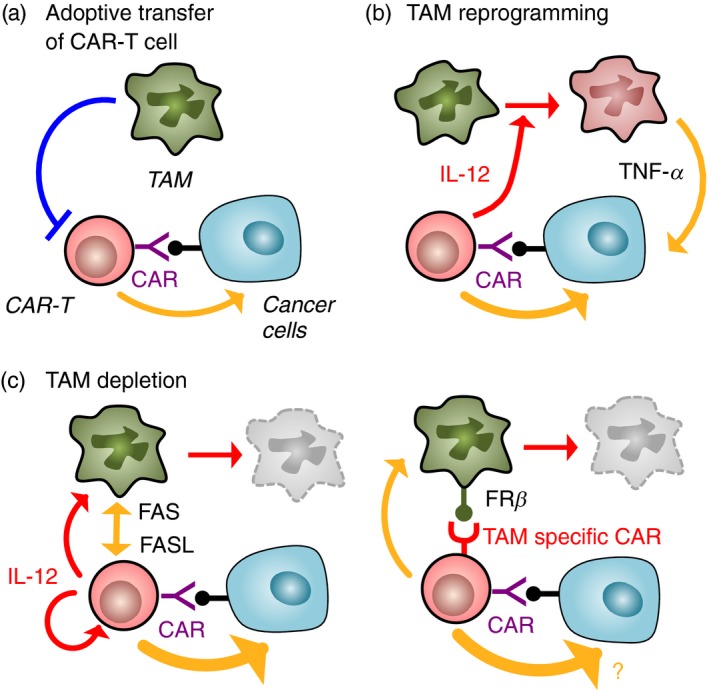
Improvement of chimeric antigen receptor T (CAR‐T) cell transfer therapy via the removal of tumour‐associated macrophages (TAM) ‐mediated immune suppression. T cells manipulated to express CAR‐T recognize surface protein on cancer cells and exert tumour‐killing activity without prior activation, whereas their functions are restricted by TAM (a). It is reported that genetic manipulation of CAR‐T cells to secrete interleukin‐12 (IL‐12) enhances efficacy of the therapy via reprogramming of TAM to tumoricidal macrophages that express tumour necrosis factor‐*α* (TNF‐*α*) (b). In another model, expression of IL‐12 in CAR‐T cells enhances therapeutic potential of CAR‐T cells by depletion of TAM as well as augmentation of cytotoxic capacity of CAR‐T cells (c; left). It is also suggested that expression of CAR against macrophage surface protein (e.g. folate receptor *β*: FR*β*) can deplete TAM, whereas its therapeutic impact on CAR‐T cell cytotoxicity against tumour cells is still not known. The yellow arrow represents cytotoxicity of CD8^+^ T cells or reprogrammed macrophages.

As mentioned above, cancer cells in malignant solid tumours often lose the expression of MHC‐I that is essential for antigen presentation to CD8^+^ T cells.^57^ As cancer cells lacking neoantigens or MHC‐I cannot be recognized by pre‐existing tumour‐infiltrating CD8^+^ T cells, therapeutic strategies aimed at boosting antigen‐dependent T‐cell activation (i.e. vaccination and checkpoint inhibition) may not be suitable for these types of tumours.^58^ One of the emerging strategies to target the non‐immunogenic cancer cells is an adoptive transfer of CD8^+^ T cells that are manipulated to express chimeric antigen receptors (i.e. CAR‐T cells).^59^ Chimeric antigen receptor (CAR) is a genetically engineered T‐cell receptor where the intracellular signalling domain is activated by extracellular single‐chain variable fragments upon direct binding to a specific protein expressed on the surface of tumour cells. Therefore, CAR‐T cells can exert cytotoxicity against tumour cells expressing the target surface molecules without MHC‐I‐restricted antigen presentation.^54^, ^60^ The CAR‐T‐cell transfer therapy has been successful for B lymphoma, but its applicability to solid tumours is still under investigation.^61^ In the solid tumours, the adoptively transferred CAR‐T cells would need to overcome the immune suppressive tumour microenvironment, including TAM in it, to exercise their therapeutic potential (Fig. 4a).

It is suggested that this requirement can be fulfilled by further genetic manipulation of CAR‐T cells to express stimulatory ligands or cytokines. In a subcutaneous tumour model, adoptive transfer of carcinoembryonic antigen (CEA) ‐specific CAR‐T cells modestly suppresses tumour growth of CEA‐expressing C15A3 cancer cells but not their parental CEA‐negative MC38 cells.^62^ In this model, co‐expression of IL‐12 in the CAR‐T cells dramatically enhances the suppressive effects of transferred CAR‐T cells on the C15A3 tumour. In the C15A3 tumour, the transfer of IL‐12‐producing CAR‐T cells but not the normal CAR‐T cells increases the number of macrophages that express TNF‐*α* in the tumours. Interestingly, the IL‐12‐producing CAR‐T cells can also inhibit tumour growth of CEA‐negative MC38 cells when these cancer cells are co‐injected with CEA‐positive C15A3 cells, and such suppressive effects disappear with anti‐TNF‐*α*‐neutralizing antibody treatment. Furthermore, cultured macrophages stimulated with IL‐12 directly kill MC38 cancer cells via production of TNF‐*α*.^62^ It has been reported that systemic injection of IL‐12 into the LLC tumour‐bearing mice significantly reduces IL‐10 and TGF‐*β* expression in TAM and concomitantly increases expression IL‐6 and TNF‐*α*.^63^ These results therefore collectively suggest that IL‐12 from CAR‐T cells reprogram macrophages recruited into the tumours to be tumoricidal cells that co‐operate with CAR‐T cells for elimination of cancer cells (Fig. 4b).

On the other hand, IL‐12 has been shown to stimulate antitumour responses through activation of CD8^+^ T cells in solid tumour models.^64^ In mice with abdominal cancer developed by ID8 ovarian cancer cells that express ectodomain of mucin‐16 (Muc16^ecto^), intraperitoneal injection of Muc16^ecto^‐specific CAR‐T cells modified to secrete IL‐12 prolongs the survival of tumour‐bearing mice.^65^ As this therapeutic effect is not observed in mice treated with IL‐12‐expressing CAR‐T cells developed from IL‐12 ‘receptor’ knockout mice, IL‐12 secreted from modified CAR‐T cells seems to act in an autocrine manner. Indeed, the Muc16^ecto^‐specific CAR‐T cells increase cytokine secretion, proliferation, cytotoxicity and survival *in vitro* when they are modified to secrete IL‐12. In this ovarian cancer model, IL‐12‐expressing CAR‐T cells increase their expression of first apoptosis signal ligand (FASL) and up‐regulate FAS expression on TAM and thereby eliminate TAM in the ascites via the FAS/FASL apoptosis pathway. As macrophage depletion by clodronate treatment significantly prolongs overall survival of tumour‐bearing mice treated with the IL‐12‐expressing CAR‐T cells, these results suggest that co‐expression of IL‐12 intensifies the therapeutic potential of CAR‐T cells by depletion of TAM in addition to the augmentation of CAR‐T‐cell cytotoxic capacity (Fig. 4c).^65^ Although IL‐12 armoured CAR‐T cells deplete TAM via an indirect mechanism,^65^ it is suggested that genetic manipulation of T cells to express TAM‐specific CAR can directly eliminate TAM in the tumours. In the ID8 ovarian cancer model, TAM in peritoneal tumour ascites expresses a high level of folate receptor *β* (FR*β*) and adoptive transfer of FR*β*‐specific CAR‐T cells into the tumour‐bearing mice results in destruction of TAMs and delayed tumour growth (Fig. 4c).^66^ However, further studies are needed to determine whether co‐expression of two different CARs, that is one for TAM and another for cancer cells, enhances therapeutic effects of CAR‐T‐cell transfer therapy. All of these studies suggest that future CAR‐T‐cell engineering should focus not only to target cancer cells but also to modulate the phenotype of TAM by their reprogramming or by their selective depletion in the tumour microenvironment.

Although therapeutic effects of TAM targeting on NK cell infusion therapy are not clear, suppressive effects of TAM on NK cell functions have been reported. For example, high infiltration of TAM in the tumours correlates with low number of interferon‐*γ*‐expressing active NK cells in human hepatocellular carcinoma and TAM from the tumour reduce activation and survival of NK cells.^17^ It is also reported that TAM isolated from the PyMT mouse mammary tumours significantly reduces cytotoxicity of NK cells *in vitro*.^18^ These results suggest that TAM depletion by blocking TAM recruitment or survival signals (e.g. treatment with CCR2 or CSF1R antagonist) can improve the efficacy of NK cell infusion therapy. After the encouraging results of CAR‐T‐cell transfer therapy, genetic engineering of NK cells has been tested at preclinical stage.^67^ Further development of this technology may enable us to manipulate NK cells to express CAR that binds to TAM (e.g. FR*β*) or TAM regulatory cytokines (e.g. IL‐12), which would enhance NK cell cytotoxicity in the solid tumours by depletion or reprogramming of TAM.

## Perspective

Cancer immunotherapy is currently being tested in several clinical trials in combination with standard therapies, and preclinical research is also active to optimize better ways to stimulate the immune system in combination with pre‐existing or novel therapies.^68^, ^69^ Unfortunately, however, not all the strategies work for all tumours and not all patients seem to respond even in those cancer subtypes where the therapy works. This is caused by different aspects such as the immunogenic properties of the tumour but also by the tumour microenvironment composition. It is becoming clear that boosting the antitumour abilities of CTL is not sufficient to exert significant tumour depletion and that a certain level of intervention in the immune suppressive tumour microenvironment must accompany the CTL activation. As summarized in this review, different studies have indicated that TAM targeting is able to synergistically enhance the response to almost all immunotherapies. Although most TAM‐targeting therapies are still at the preclinical stage, antagonists or blocking antibodies that can be used for TAM depletion (e.g. CCR2 or CSF1R antagonists) have already been tested in clinical trials for malignant solid tumours.^70^, ^71^ Further investigation of synergistic effects of these agents with immunotherapies will lead to the improvement of ongoing immunotherapeutic strategies. On the other hand, data suggest that macrophages in the solid tumours can engage a robust antitumour immune response in some cases and so TAM depletion is not necessarily associated with good outcome in some immunotherapies such as cancer vaccination. Therefore, deciphering the exact molecular mechanisms responsible for macrophage polarization during treatment is necessary to determine effective TAM‐targeting approaches to improve immunotherapies. It is also necessary to identify specific markers and immune suppressive molecules of TAM in different cancers and cancer subtypes for the targeting of only the tumour‐promoting macrophage subpopulations. Results from these basic studies will be helpful to choose the appropriate TAM‐targeting strategy (depletion, reprogramming and molecular targeting) and tailor it based on the type of administered immunotherapy.

## Disclosures

The authors declare no conflict of interests.
